# Dose-related depressive symptoms and suicidal ideation during cabergoline therapy in an adolescent with macroprolactinoma

**DOI:** 10.1210/jcemcr/luag351

**Published:** 2026-11-06

**Authors:** Saniha Malik, Amira Al Harrasi, Maryam Issa Al Kiyumi, Maisa Hamed Al Kiyumi

**Affiliations:** Primary Health Care, Ministry of Health, Muscat 100, Oman; Family Medicine and Public Health, Sultan Qaboos University Hospital, University Medical City, Muscat 123, Oman; Family Medicine and Public Health, College of Medicine and Health Sciences, Sultan Qaboos University, Muscat 123, Oman; Family Medicine and Public Health, Sultan Qaboos University Hospital, University Medical City, Muscat 123, Oman; Family Medicine and Public Health, College of Medicine and Health Sciences, Sultan Qaboos University, Muscat 123, Oman; Family Medicine and Public Health, Sultan Qaboos University Hospital, University Medical City, Muscat 123, Oman; Family Medicine and Public Health, College of Medicine and Health Sciences, Sultan Qaboos University, Muscat 123, Oman

**Keywords:** cabergoline, macroprolactinoma, adolescent, depressive symptoms, suicidal ideation, dopamine agonist

## Abstract

Cabergoline is the preferred medical treatment for prolactinomas in children and adolescents because of its efficacy in reducing prolactin concentrations and tumor size. Although dopamine agonists are generally well tolerated, neuropsychiatric adverse effects have been reported, with depressive symptoms and suicidal ideation less frequently described. We report a 15-year-old girl with macroprolactinoma who developed low mood, emotional dysregulation, crying spells, reduced appetite, sleep–wake disturbance, loss of interest in previously enjoyable activities, and passive suicidal thoughts after cabergoline was increased to 1 mg twice weekly. She had no previous psychiatric history, major psychosocial stressor, manic symptoms, or psychotic features. Psychiatric assessment identified moderate depressive symptoms, with a Patient Health Questionnaire-9 score of 11. Cabergoline was reduced to 0.75 mg twice weekly because of the close temporal relationship between dose escalation and symptom onset. Her psychiatric symptoms almost completely resolved within a few days, while prolactin remained controlled. The temporal association, apparent dose relationship, and rapid improvement following dose reduction suggest a cabergoline-associated neuropsychiatric adverse reaction. Clinicians should monitor mood and suicidal ideation during dopamine agonist therapy, particularly after dose escalation and in adolescents.

## Introduction

Macroprolactinomas are uncommon in children and adolescents and may present with endocrine dysfunction and, in larger tumors, mass effects. Their management is particularly important during adolescence because prolonged hyperprolactinemia can interfere with pubertal development and gonadal function. Dopamine agonists are the first-line medical treatment for pediatric and adolescent prolactinomas, with cabergoline generally preferred because of its efficacy in lowering prolactin concentrations and reducing tumor size [1, 2].

Cabergoline is generally well tolerated, but its effects on central dopaminergic pathways have been associated with a range of neuropsychiatric adverse effects. Published reports have described impulse-control disorders (ICDs), mood instability, mania, psychosis, behavioral disturbances, and, less commonly, depressive symptoms and suicidal ideation [3-8]. Impulse-control disorders have received particular attention in patients with prolactinomas, whereas predominantly depressive presentations remain relatively uncommon in the published literature [9-11].

Recognition of treatment-related psychiatric symptoms may be challenging, particularly in adolescents, in whom changes in mood and behavior may initially be attributed to developmental or psychosocial factors. We describe an adolescent with macroprolactinoma who developed depressive symptoms, emotional dysregulation, and suicidal ideation following the escalation of cabergoline therapy, with marked improvement shortly after dose reduction. The temporal and dose-related pattern of symptoms provides an important clinical consideration when monitoring adolescents receiving dopamine agonist therapy.

## Case presentation

A previously healthy 15-year-old girl was referred to the endocrine service for evaluation of secondary amenorrhea. She had attained menarche at 11 years of age, followed by irregular menstrual cycles. After an initial 6-day menstrual period, she experienced 2 months of amenorrhea followed by another 6-day period. Her last menstrual period occurred in November 2024, after which she remained amenorrheic. Menstrual flow had generally been scant. She denied cyclical abdominal or pelvic pain, breast tenderness, galactorrhea, acne, or hirsutism. She reported weight gain but had no dysmorphic features. She denied any history of headaches or visual disturbances, including blurred vision, diplopia, or visual field defects. Her growth and developmental history were unremarkable.

Her medical history was otherwise unremarkable apart from mild bilateral myopia, for which she was followed by ophthalmology. An aunt had a history of polycystic ovarian syndrome; there was no known family history of breast or ovarian disease. She was clinically well, with normal vital signs and a soft, nontender abdomen.

## Diagnostic assessment

Subsequent endocrine evaluation identified marked hyperprolactinemia. Serum prolactin concentrations were 599.5 ng/mL (SI: 12 709 mIU/L) and 620.4 ng/mL (SI: 13 152 mIU/L) on two separate measurements in September 2025 (reference range, 5.2-26.5 ng/mL [SI: 110-562 mIU/L]). The serum estradiol concentration was 27.2 pg/mL (SI: 0.1 nmol/L) (reference range, 294.2-751.9 pg/mL [SI: 1.08-2.76 nmol/L]) with follicle-stimulating hormone of 4.9 mIU/mL (SI: 4.9 IU/L) (reference range, 1.7-7.7 mIU/mL [SI: 1.7-7.7 IU/L]), and luteinizing hormone of 3.3 mIU/mL (SI: 3.3 IU/L) (reference range, 1.0-11.4 mIU/mL [SI: 1.0-11.4 IU/L]). Free thyroxine was 1.11 ng/dL (SI: 14.3 pmol/L) (reference range, 0.98-1.63 ng/dL [SI: 12.6-21.0 pmol/L]), thyroid-stimulating hormone was 1.8824 µIU/mL (SI: 1.8824 mIU/L) (reference range, 0.51-4.30 µIU/mL [SI: 0.51-4.30 mIU/L]), testosterone was 35.8 ng/dL (SI: 1.24 nmol/L) (reference range, 4.6-38.4 ng/dL [SI: 0.16-1.33 nmol/L]), 10.4 µg/dL (SI: 287 nmol/L) (reference range, 4.8-19.5 µg/dL [SI: 133-537 nmol/L]), and insulin-like growth factor (IGF-1) concentration was the serum IGF-1 concentration was 218.6 ng/mL (SI: 28.5 nmol/L) (reference range, 80.4-904.3 ng/mL [SI: 10.48-117.9 nmol/L]). Basic metabolic investigations were within normal limits. Pelvic ultrasonography performed during the initial evaluation showed a small uterus measuring 25 × 16 × 26 mm, with an endometrial thickness of 1 mm and apparently normal ovaries bilaterally.

Pituitary magnetic resonance imaging demonstrated a 14-mm sellar lesion with solid and cystic components extending into the suprasellar compartment and closely abutting the optic chiasm, without frank chiasmal compression. Confrontation visual-field testing was normal. The findings were consistent with a macroprolactinoma associated with hypogonadotropic hypogonadism and secondary amenorrhea. Given the patient's young age at presentation, genetic predisposition syndromes, including aryl hydrocarbon receptor-interacting protein-related pituitary adenoma and multiple endocrine neoplasia type 1, were considered. The patient was referred for genetic evaluation; however, consultation and genetic testing are still pending.

## Treatment

Cabergoline was initiated at a dose of 0.25 mg twice weekly and was subsequently titrated according to the biochemical response. Approximately 2 months after treatment initiation, the serum prolactin concentration had decreased to 68.0 ng/mL (SI: 1441 mIU/L), and the dose was increased to 0.5 mg twice weekly. Approximately 2 months later, prolactin had decreased further to 38.3 ng/mL (SI: 813 mIU/L), prompting a further increase to 0.75 mg twice weekly. After a further 2 months of treatment, the prolactin concentration had decreased to 26.9 ng/mL (SI: 570 mIU/L), and cabergoline was increased to 1 mg twice weekly. Overall, the treatment produced a substantial biochemical response, with prolactin decreasing from 620.4 ng/mL (SI: 13 152 mIU/L) to 26.9 ng/mL (SI: 570 mIU/L) and resumption of regular menstrual cycles.

Within 1 month of escalation to 1 mg twice weekly, the patient developed prominent depressive and behavioral symptoms, including persistent low mood, frequent crying, guilt, reduced appetite, sleep–wake disturbance, reduced school attendance, loss of interest in drawing, and increased irritability. She also reported occasional passive thoughts of death and 1 episode of self-injurious behavior following an argument with a friend. She noted that the symptoms were more pronounced on the days immediately following cabergoline administration.

She was assessed by her general practitioner while accompanied by her father. There was no evidence of psychosis or mania, and she denied active suicidal ideation at the time of assessment. Her Patient Health Questionnaire-9 (PHQ-9) score was 11, consistent with moderate depressive symptoms.

Given the close temporal relationship between the dose escalation and onset of symptoms and the reported worsening after medication administration, cabergoline-associated neuropsychiatric adverse effects were considered. After discussion with the endocrinology team, the cabergoline dose was reduced from 1 mg to 0.75 mg twice weekly rather than discontinued, given the substantial biochemical response to treatment. Repeat prolactin measurements were performed after 4 weeks, which remained well controlled at 9.6 ng/mL (SI: 203 mIU/L).

## Outcome and follow-up

Following reduction of cabergoline from 1 mg to 0.75 mg twice weekly, the patient's depressive symptoms and emotional dysregulation improved markedly within a few days and almost completely resolved. Her repeated PHQ-9 score was 3. The improvement occurred without complete discontinuation of cabergoline or initiation of specific psychiatric pharmacotherapy. This clinical response was accompanied by continued biochemical control of the prolactinoma, with a subsequent serum prolactin concentration of 9.6 ng/mL (SI: 203 mIU/L).

No recurrence of the previously reported depressive symptoms or suicidal ideation was documented following dose reduction. The patient remained under endocrinology follow-up, with continued monitoring of prolactin concentrations and clinical response to the reduced cabergoline dose. No additional adverse or unanticipated events were reported during the documented follow-up period.

The temporal pattern showed that psychiatric symptoms emerged after cabergoline dose escalation and rapidly improved after dose reduction while treatment was maintained, representing a positive dechallenge and supporting a possible dose-related association between cabergoline exposure and the patient's neuropsychiatric symptoms.

## Discussion

Neuropsychiatric adverse effects of dopamine agonists are increasingly recognized in patients treated for prolactinomas. The published literature, however, has focused predominantly on ICDs, including hypersexuality, pathological gambling, compulsive shopping, and binge eating. In a multicenter study of 308 patients with prolactinoma receiving dopamine agonists, Dogansen et al [9] reported ICDs in 17% of patients, with hypersexuality being the most frequently identified manifestation. A prospective study similarly demonstrated that new-onset ICDs can occur during dopamine agonist therapy, with symptoms improving or resolving after dose reduction or discontinuation [10]. Reviews of the available literature have further highlighted the occurrence of ICDs across patients receiving dopamine agonists for hyperprolactinemia [11, 12]. In contrast, predominantly depressive reactions accompanied by suicidal ideation appear to be less frequently reported.

The present case is notable for the predominance of depressive and affective symptoms following escalation of cabergoline in an adolescent with no documented previous psychiatric disorder. The patient developed low mood, frequent crying, excessive guilt, reduced appetite, sleep–wake disturbance, loss of interest in previously enjoyable activities, irritability, and passive thoughts of death within 1 month of increasing cabergoline to 1 mg twice weekly. There were no psychotic or manic features, and no major psychosocial stressor was identified before symptom onset. The clinical presentation therefore differed from the impulse-control and behavioral activation syndromes more commonly associated with dopamine agonist therapy.

Several published reports provide relevant comparisons. Table 1 summarizes these published cases. Mignone et al [4] described a patient with giant prolactinoma who developed insomnia, emotional lability, low mood, lethargy, and intermittent suicidality while receiving cabergoline 500 mcg twice weekly. Psychiatric symptoms were considered related to dopamine agonist therapy, and a lower cabergoline dose was subsequently tolerated without comparable psychological adverse effects. Similarly, Dravid et al [13] reported major depression with atypical features in a 28-year-old woman receiving cabergoline, with improvement after dose reduction and treatment with sertraline. In another report, Burback [3] described depressed mood and cyclical mood disturbance during cabergoline treatment that subsequently progressed to mania with psychotic features. Davie [7] reported major depression and paranoid symptoms in a patient with prolactinoma receiving long-term cabergoline, although pathological gambling was the predominant treatment-emergent behavioral manifestation. Importantly, data suggest that hyperprolactinemia itself is linked to increased rate of psychological disturbances, including anxiety and depression [14]. The hypothesized mechanism was not fully understood and probably ascribed to the prolactin-induced suppression of the hypothalamic–pituitary–gonadal axis in addition to the direct effect of prolactin levels on brain functions [14].

**Table 1:** Published reports of depressive and affective symptoms associated with cabergoline therapy

Patient/Clinical context	Cabergoline exposure	Psychiatric presentation	Management and outcome	Ref.
32-year-old woman with microprolactinoma	Initially 0.25 mg/week, increased to 0.25 mg twice weekly	Depressed mood and cyclical mood disturbance, subsequently progressing to mania with psychotic features	Cabergoline discontinued and aripiprazole initiated; manic and psychotic symptoms resolved	[3]
Patient with giant prolactinoma	0.5 mg twice weekly	Insomnia, emotional lability, low mood, lethargy, and intermittent suicidal ideation	Psychiatric review and escalation of escitalopram; cabergoline was subsequently reduced, with the lower dose tolerated without comparable psychological adverse effects	[4]
47-year-old woman with prolactinoma	0.25 mg weekly	Pathological gambling accompanied by major depression and paranoid delusions	Cabergoline discontinued and escitalopram initiated; mood and psychotic symptoms improved and pathological gambling ceased	[7]
28-year-old woman with prolactinoma	Restarted at 0.5 mg; frequency not reported	Irritability, hypersomnia, fatigue, and crying spells; diagnosed with major depression with atypical features	Cabergoline dose reduced and sertraline initiated; significant clinical improvement was reported	[13]

The temporal pattern in the present case provides additional support for a possible association with cabergoline exposure. Symptoms emerged shortly after the dose was increased to 1 mg twice weekly and substantially improved within a few days after the reduction to 0.75 mg twice weekly. This finding constitutes a positive dechallenge. Importantly, cabergoline was not discontinued, allowing continued treatment of the macroprolactinoma while the psychiatric symptoms improved. A similar response to dose reduction has been described in other reports of cabergoline-associated psychiatric adverse effects [4, 15-17]. Nevertheless, a positive dechallenge alone does not establish causality, and alternative explanations for depressive symptoms cannot be completely excluded in an individual adolescent.

The broader literature demonstrates that cabergoline-associated neuropsychiatric manifestations are heterogeneous. Psychosis, mania, delirium, hallucinations, aggressive behavior, and compulsive behaviors have all been reported [3, 5, 6]. Several cases have demonstrated improvement following dose reduction or discontinuation, including recurrence after rechallenge in at least one report, providing additional evidence for an exposure-related relationship [17]. The present case extends this spectrum by describing a predominantly depressive presentation with suicidal ideation without psychosis or mania. The rapid improvement following a relatively modest dose reduction is also clinically relevant because it suggests that dose adjustment may be sufficient in selected patients while maintaining dopamine agonist therapy.

The mechanism underlying depressive symptoms during cabergoline treatment remains uncertain. Cabergoline produces potent stimulation of dopamine D2 receptors, and alterations in central dopaminergic signaling may influence mood, motivation, reward processing, and behavior. However, the occurrence of depressive symptoms alongside a drug with predominantly dopaminergic activity suggests that the neuropsychiatric effects of dopamine agonists cannot be reduced to a simple model of behavioral activation. Individual susceptibility, receptor distribution, underlying neurobiology, and developmental factors may contribute to the variability of clinical responses [3, 5].

The adolescent age of the patient is particularly relevant. Changes in mood, sleep, behavior, and social functioning during adolescence may have multiple potential explanations, which can make recognition of medication-related adverse effects challenging. In this case, the close temporal relationship between dose escalation and symptom onset, the reported worsening after medication administration, absence of a clear preceding psychosocial trigger, and rapid improvement following dose reduction collectively supported consideration of cabergoline as a contributing factor. Clinicians caring for adolescents receiving dopamine agonists should therefore inquire about mood changes, sleep disturbance, behavioral changes, impulsivity, and suicidal thoughts, particularly following initiation or dose escalation. Patients and caregivers should be encouraged to report clinically significant affective or behavioral changes promptly.

This case has several limitations. First, causality cannot be established from a single case, and unrecognized psychosocial or biological factors may have contributed to the symptoms. Second, the follow-up period documented in the available clinical record is limited, restricting assessment of long-term recurrence or sustained psychiatric and biochemical outcomes. Third, formal psychiatric assessment was performed after symptom onset rather than prospectively before cabergoline initiation, limiting comparison with the patient's pretreatment psychiatric state. Fourth, the available pediatric literature on cabergoline-associated depressive symptoms and suicidal ideation remains sparse, making it difficult to determine the incidence, dose–response relationship, or specific risk factors for this adverse effect. Finally, it is important to highlight the onset–offset asymmetry of psychiatric symptoms in the current case, in which the symptoms developed over approximately 1 month but almost resolved within 3 days of cabergoline dose reduction, despite the cabergoline prolonged elimination half-life. A possible explanation is a threshold-dependent effects of dopamine on neuropsychiatric function. However, it is essential to note that rapid resolution of symptoms could be coincidental and therefore causal relationship cannot be inferred.

Further longitudinal studies are needed to characterize the frequency, clinical spectrum, dose relationship, and potential risk factors for neuropsychiatric adverse effects during dopamine agonist treatment in children and adolescents. Until such evidence is available, awareness and targeted monitoring for affective and behavioral changes may help facilitate early recognition and appropriate dose adjustment.

## Learning points

- Cabergoline-associated neuropsychiatric adverse effects may include depressive symptoms and suicidal ideation in addition to the more widely recognized impulse-control disorders, mania, and psychosis.
- New-onset mood or behavioral changes in adolescents receiving cabergoline should prompt assessment for a possible medication-related adverse effect, particularly following dose escalation.
- A close temporal relationship between dose escalation and symptom onset, followed by rapid improvement after dose reduction, may support a dose-related association, although it does not establish causality.
- In selected patients, reducing the cabergoline dose rather than discontinuing treatment may alleviate neuropsychiatric symptoms while allowing continued treatment of prolactinoma.
- Adolescents and their caregivers should be counseled to promptly report significant changes in mood, behavior, sleep, or suicidal thoughts during dopamine agonist therapy.

## Data Availability

No genomic or sequence datasets were generated or analyzed in this case report. Relevant clinical data are presented within the manuscript. Data sharing is not applicable to this article, as no datasets were generated or analyzed during the current study.
